# Biallelic *ERBB3* loss-of-function variants are associated with a novel multisystem syndrome without congenital contracture

**DOI:** 10.1186/s13023-019-1241-z

**Published:** 2019-11-21

**Authors:** Niu Li, Yufei Xu, Yi Zhang, Guoqiang Li, Tingting Yu, Ruen Yao, YunFang Zhou, Yiping Shen, Lei Yin, Xiumin Wang, Jian Wang

**Affiliations:** 1 Department of Medical Genetics and Molecular Diagnostic Laboratory, Shanghai Children’s Medical Center, Shanghai Jiaotong University School of Medicine, 1678 Dongfang Road, Shanghai, 200127 People’s Republic of China; 2 Institute of Pediatric Translational Medicine, Shanghai Children’s Medical Center, Shanghai Jiaotong University School of Medicine, 1678 Dongfang Road, Shanghai, 200127 People’s Republic of China; 3 Department of Pediatrics, Shanghai Children’s Medical Center, Shanghai Jiaotong University School of Medicine, Shanghai, 200127 China; 4 Division of Genetics and Genomics, Boston Children’s Hospital, Harvard Medical School, Boston, MA 02115 USA; 5 Department of Endocrinology and Metabolism, Shanghai Children’s Medical Center, Shanghai Jiaotong University School of Medicine, Shanghai, 200127 China

**Keywords:** ERBB3 gene, Novel compound heterozygous variants, Loss of function, Novel multisystem syndrome, Functional study

## Abstract

**Background:**

Gain-of-function pathogenic variants of the Erb-B2 receptor tyrosine kinase 3 (*ERBB3*) gene contribute to the occurrence and development of a variety of human carcinomas through activation of phosphatidylinositol 3-kinase (PI3K)/AKT and extracellular signal-regulated kinase (ERK) signaling. ERBB3 gene homozygous germline variants, whose loss of function may cause autosomal recessive congenital contractural syndrome, were recently identified. This study aims to identify the disease-causing gene in a Chinese pedigree with variable phenotypes involving multiple systems, including developmental delay, postnatal growth retardation, transient lower limb asymmetry, facial malformations, atrioventricular canal malformation, bilateral nystagmus and amblyopia, feeding difficulties, immunodeficiency, anemia, and liver damage, but without congenital contracture.

**Methods:**

Trio-whole exome sequencing (WES) was performed to identify the disease-causing gene in a 24-month-old Chinese female patient. The pathogenicity of the identified variants was evaluated using in silico tools and in vitro functional studies.

**Results:**

Trio-WES revealed compound heterozygous variants of c.1253 T > C (p.I418T) and c.3182dupA (p.N1061Kfs*16) in the *ERBB3* gene. Functional studies showed that p.I418T resulted in normal expression of ERBB3, which was capable of interacting with ERBB2. However, the variant impaired ERBB3 phosphorylation, consequently blocking ERBB2 phosphorylation and AKT and ERK activation. The truncated protein resulting from the c.3182dupA variant also lacked the capacity to activate downstream signaling pathways.

**Conclusions:**

We report the first patient with a novel multisystem syndrome disorder without congenital contracture resulting from biallelic loss-of-function variants of *ERBB3*.

## Background

The Erb-B2 receptor tyrosine kinase (ERBB)/HER family of receptor tyrosine kinases includes four transmembrane glycoprotein epidermal growth factor receptors (EGFR/HER1, ErbB2/HER2, ErbB3/HER3, and ErbB4/HER4) that play essential roles in the regulation of cell survival, proliferation, adhesion, and differentiation through activation of phosphatidylinositol 3-kinase (PI3K)/AKT and extracellular signal-regulated kinase (ERK) signaling pathways [1, 2]. The *ERBB3* gene (NM_001982.3) is located on 12q13.2 and encodes a 1342-amino acid protein that is widely expressed in various tissues in humans and mice. The ERBB3 protein has a heregulin (HRG)-binding domain and can bind to and is phosphorylated by neuregulin (NRG)1 depending on interaction with integrins [3]. However, phosphorylated ERBB3 cannot directly transmit intracellular signals since it lacks an active kinase domain; instead, it activates downstream pathways via heterotypic interactions with other members of the EGFR family, with the ERBB2/ERBB3 heterodimer eliciting the strongest downstream response [4]. Somatic heterozygous activating mutations of the *ERBB3* gene have been linked to the occurrence of numerous solid tumors including those of the prostate, bladder, gall bladder, colon, gastric, and breast [5–7]. In addition, a heterozygous germline activating variant of the ERBB3 gene has been implicated in myelodysplastic syndromes [8].

In 2003, 23 cases of autosomal recessive congenital contractural syndrome (OMIM#607598) were reported in a large Israeli Bedouin kindred pedigree; the phenotype included multiple joint contractures, anterior horn atrophy in the spinal cord, and an unusual distended urinary bladder [9]. Subsequent work identified a homozygous germline splice variant (c.1184-9A > G) of the *ERBB3* gene in two pedigrees that was predicted to produce a frameshift variant (p.G395Afs*4) [10, 11]. This was the first report of a human phenotype that might result from loss of function of the *ERBB3* gene. Recently, a homozygous missense variant (c.3425C > T; p.P1142L) of *ERBB3* was reported in a patient with knee dislocation and bilateral hip dislocation [12]. However, due to the limitation of the number of cases and the lack of definite evidence from functional studies, there is still insufficient evidence of a link between germline loss-of-function variants of the *ERBB3* gene and Mendelian phenotypes.

Here, we report a 24-month old Chinese female patient presenting with a novel multisystem syndrome, sharing the features of developmental delay, postnatal growth retardation, transient lower limb asymmetry, facial malformations, atrioventricular canal malformation, bilateral nystagmus and amblyopia, feeding difficulties, immunodeficiency, anemia, and liver damage. Trio-whole exome sequencing (WES) identified compound heterozygous variants (c.1253 T > C;p.I418T and c.3182dupA;p.N1061Kfs*16) of the *ERBB3* gene. In vitro functional analyses of the two variants revealed that they abolished ERBB2/ERBB3 phosphorylation, leading to failure to activate downstream AKT and ERK signaling pathways.

## Methods

### Patient description

The proband was a 24-month-old Chinese girl born to physically healthy and non-consanguineous parents at 40 ^+ 6^ weeks with a birth weight of 2700 g (− 1.4 SD) and a birth length of 47.5 cm (− 1.4 SD) by Caesarean section delivery due to fetal intrauterine distress. The patient was the couple’s first child, and the mother had a prior miscarriage at 3 months for unknown reasons. After birth, the patient experienced feeding difficulties and recurrent respiratory infection, for which she was hospitalized several times. She could not open her eyes until 1 month of age and did not pass the visual inspection test when she underwent ophthalmological examination. She was suspected of having weakened immune function because of IgA deficiency (0.30 g/l; reference range: 0.7–4.0 g/l), IgG deficiency (4.5 g/l; reference range: 7–16 g/l), and recurrent respiratory infection, and hence, received infusion of gamma globulin at 3 months of age.

**Fig. 1:**
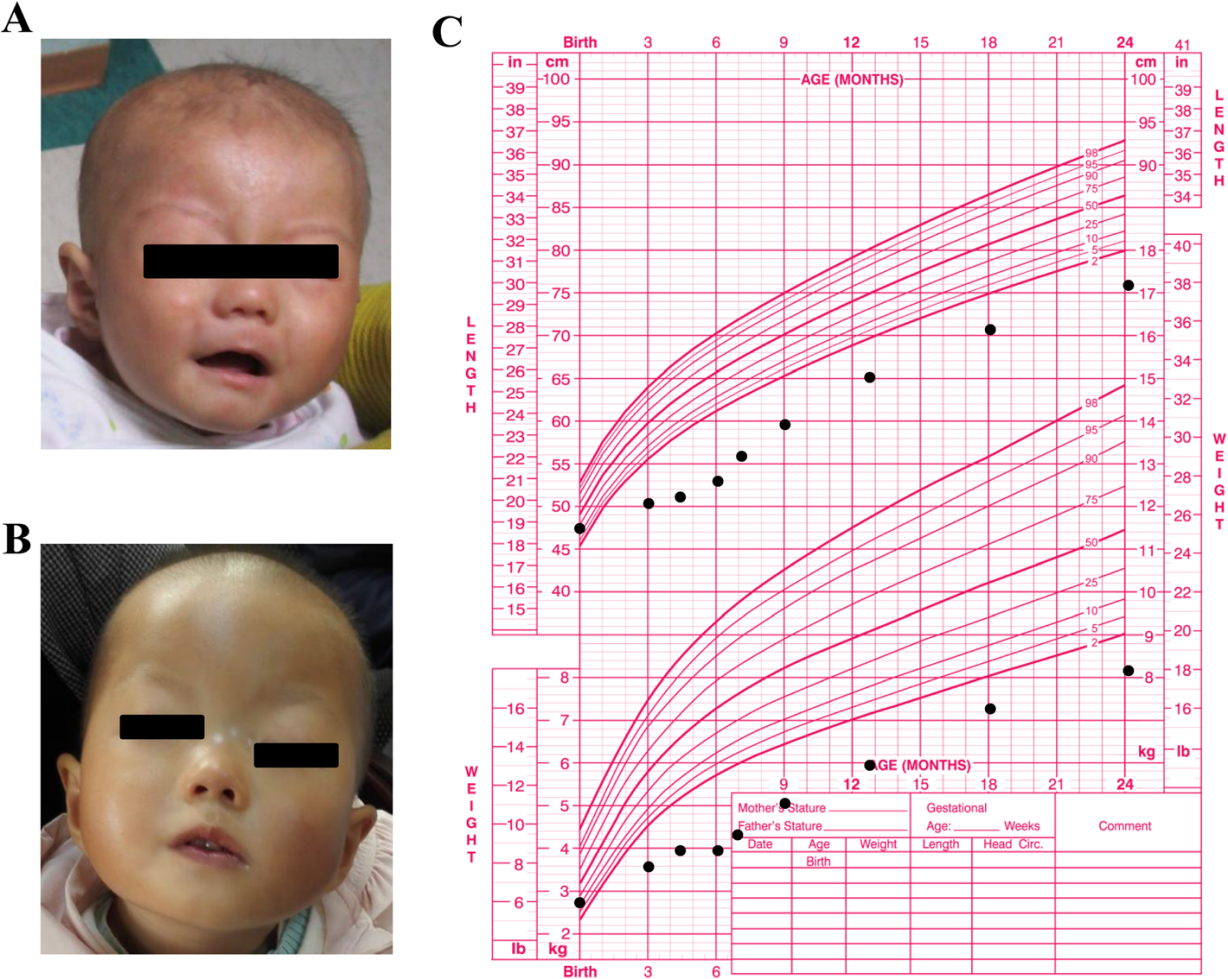
The patient’s clinical features. **a**, **b** Facial malformations of the patient at 7 months of age (**a**) and 2 years of age (**b**). **c** The patient’s growth chart from birth to 24 months of age

At 7 months of age, the patient showed development delay, low weight (4.2 kg, − 4.3SD), and short stature (56 cm, − 5.2 SD). She was unable to raise and steady her head or turn over while lying down. She had sparse hair, ptosis, depressed nasal bridge, and the right corner of her mouth was oblique (Fig. 1a and b, Table 1). Her eyes did not fully open. Her left lower limb was 0.9 cm shorter than the right lower limb, and the left foot was smaller than the right foot. Cardiac ultrasound results showed atrioventricular canal malformation. Blood biochemical analyses showed elevated lactate dehydrogenase (1443 U/l; reference range: 187–367 U/l), aspartate aminotransferase (217 U/l; reference range: 15–46 U/l), and alanine aminotransferase (307 U/l; reference range: 13–69 U/l) levels, which eventually normalized after treatment with reduced glutathione (GSH). She had normal levels of immunoglobulin (Ig) G, IgE, complement (C)3, and C4 but IgA deficiency (< 0.26 g/l). The routine blood test revealed anemia (hemoglobin, 88 g/l; reference range: 110–160 g/l). The findings of the cranial magnetic resonance imaging, ultrasound of the abdomen and thyroid, arterial blood gas test, blood and urinary tandem mass spectrometry, and chromosome karyotyping were normal. The patient underwent partial atrioventricular canal (PAVC) correction surgery at 1 year old.

**Table 1:** Clinical features of patients with biallelic *ERBB3* mutations

	This study	Landau et al.(Ref. [9])	Alfares et al. (Ref. [12])
Variant	c.1253 T > C; p.Ile418Thr (Het) and c.3182dupA; p.Asn1061Lysfs*16 (Het)	c.1184-9A > G (Hom)	c.3425C > T; p.Pro1142Leu (Hom)
No. of patients	1	23	1
Birth conditions	Fetal intrauterine distress, normal birth weight	All newborns were small or borderline adequate for gestational age	No data
Facial malformations	High forehead, sparse hair and eyebrow, hypertelorism, ptosis, depressed nasal bridge, bulbous nasal tip, low-set ears, right oblique corner of the mouth, micrognathia	8 cases with micrognathia, 2 cases with cleft palate, and one with anisocoria	No data
Skeletal deformities			
Multiple joint contracture	–	23/23	No data
Hip dislocation	–	2/23	+
Knee dislocation	–	–	+
Asymmetrical limbs	+	–	No data
Kidney malformation	–	Hydronephrosis and cystic changes of the kidneys in 5 cases	No data
Enlarged urinary bladder	–	12/23	No data
Cardiac malformation	Atrioventricular canal malformation	2 cases with VSD and one with dilated cardiomyopathy	No data
Ophthalmologic problems	Nystagmus, amblyopia	High myopia and degenerative vitreoretinopathy in two patients	No data
Respiratory problems	Recurrent respiratory infection	Respiratory insufficiency in 15 cases	No data
Developmental delay	+	No data	No data
Transient liver damage	+	No data	No data
Immunodeficiency	IgA deficiency	No data	No data
Anemia	+	No data	No data
Feeding difficulties	+	No data	No data
Clinical outcome	Still alive (24-month old)	2 girls alive at 12 and 13 years old, respectively; 17 died from soon after birth to 1 year old.	No data

*Hom* Homozygote, *Het* Heterozygote, *VSD* ventricular septal defect

At 24 months, the patient’s height was 75.2 cm (− 3.9 SD); her weight was 8.05 kg (− 3.1 SD); and body mass index (BMI) was 14.2. A growth chart of length and weight was summarized to show her developmental history (Fig. 1c). Her head circumference (occipitofrontal diameter) was 44.8 cm (− 1.9 SD). A physical examination revealed a protruding forehead with sparse hair and eyebrows, ptosis, depressed nasal bridge, bulbous nasal tip, low-set ears, and micrognathia (Fig. 1b). Limb-length asymmetry had become less obvious and was difficult to detect. The patient could stand with support, but was unable to walk alone or speak. She still suffered from recurrent respiratory infection at a frequency of once per month. Ophthalmic examination revealed bilateral nystagmus and amblyopia. No abnormalities were found in joint radiography (Additional file 1: Figure S1). The family had another daughter who was 1 month old and showed no abnormalities in the pre- and postnatal examinations.

Before pregnancy, the patient’s mother was screened for TORCH, which includes toxoplasmosis, other (Hepatitis B, syphilis, varicella-zoster, parvovirus B19), rubella, cytomegalovirus (CMV), and herpes infections. All the tests results were normal. The patient was tested for Epstein-Barr virus and CMV at ages 1 month and 3 months, respectively, and all the test results were normal. Moreover, she was also negative for the hepatitis A, B, C, and E viruses at age 1 month. No relevant finding was obtained from her living environment.

### Gene sequencing

WES was performed on the samples obtained from the patient and her parents as described in our previous study [13]. Sanger sequencing for confirmation of *ERBB3* gene (NM_001982.3) variants was performed on an Applied Biosystems ABI3730XL sequencer (Thermo Fisher Scientific, Waltham, MA, USA) with forward and reverse primers for amplifying exon 11 (5′-GAAACCAAATGCTGAGGCTG-3′ and 5′-CTATGACACAGGCTTCATTGC-3′, respectively) and exon 27 (5′-CACAAACCCTACAGATACCCAG-3′ and 5′-CCTCAGAGGTTTCTAATGTCTTCC-3′). Sequencing data were analyzed using Mutation Surveyor v.4.0.4 software (SoftGenetics LLC, State College, PA, USA).

### In silico analysis of the ERBB3-p.Ile418Thr variant

Conservation analysis of the ERBB3 p.Ile418 variant was performed using MultAlin online software (http://multalin.toulouse.inra.fr/multalin/). The potential pathogenicity of the variants was assessed using VarCards (http://varcards.biols.ac.cn/) [14]. The three-dimensional (3D) structure of the wild-type (WT) ERBB3 protein was obtained from Protein Data Bank (https://www.rcsb.org/structure/1m6b) and was examined using Pymol v.1.8.4.0 software (https://www.pymol.org; Schrödinger, New York, NY, USA). The 3D structure of the mutant ERBB3 protein was generated after substituting isoleucine 418 with threonine.

#### Multiplex ligation-dependent probe amplification (MLPA)

MLPA was performed using the “SALSA MLPA probemix ME030-BWS/RSS” and “ME032-A1 UPD7/UPD14” kits (MRC Holland, Amsterdam, The Netherlands) according to the manufacturer’s protocol. Data analysis and interpretation were performed using GeneMarker software (Softgenetics, State College, PA, USA).

### Plasmid construction, cell culture, and transfection

Open reading frame sequences of WT *ERBB3* (NM_001982.3, from the start codon to the stop codon) were ligated into pCDH1-MSCV-EF1-GreenPuro cDNA Cloning and Expression Vector (System Biosciences, Palo Alto, CA, USA)—which contains a myc tag (GAGCAGAAGCTGATCTCAGAGGAGGACCTG) at the N terminus. Mutant ERBB3 expression plasmids were constructed from WT plasmids by site-directed mutagenesis using the QuikChange II XL Site-Directed Mutagenesis Kit (Agilent Technologies). The primers for mutant plasmid construction are listed in Additional file 2: Table S1. *ERBB2* cDNA (NM_004448.3, from the start codon to the stop codon) was ligated into the pcDNA3.1 expression vector containing the enhanced green fluorescent protein (GFP) sequence (#13031, Addgene, Watertown, MA, USA). All plasmids were prepared using ZymoPURE II Plasmid Midi-prep Kit (Irvine, CA, USA). HEK293T cells were grown in Dulbecco’s modified Eagle’s medium supplemented with 10% (v/v) fetal bovine serum (Thermo Fisher Scientific) and 1% penicillin/streptomycin (Thermo Fisher Scientific) in a 5% CO2 incubator at 37 °C. The cells were transfected with the plasmids using Lipofectamine 2000 (Invitrogen, Carlsbad, CA, USA) according to the manufacturer’s protocol.

### Immunoprecipitation and immunoblotting

For immunoprecipitation, at 48 h after plasmid transfection, HEK293T cells were washed twice with ice-cold phosphate-buffered saline and then lysed in Pierce immunoprecipitation lysis buffer (Thermo Fisher Scientific; #87787) with protease inhibitor for 20 min at 4 °C. Crude lysates were cleared by centrifugation at 20,000×g at 4 °C for 10 min, and the supernatant was incubated with GFP-Trap (ChromoTek, Hauppauge, NY, USA) for 2 h at 4 °C. The immunoprecipitates were extensively washed three times with lysis buffer and eluted with 2× sodium dodecyl sulfate–polyacrylamide gel electrophoresis loading buffer by boiling for 10 min. For immunoblotting, whole-cell extracts were prepared in radioimmunoprecipitation assay buffer (#R0278, Sigma-Aldrich, St. Louis, MO, USA) containing protease and phosphatase inhibitors (Thermo Fisher Scientific; 78,440). To induce protein phosphorylation, cells were treated with 20 ng/ml recombinant human NRG-1β (R&D Systems, Minneapolis, MN, USA. #396HB) for 30 min before they were harvested. Samples were subjected to SDS–PAGE and immunoblotting using standard procedures.

### Antibodies

Antibodies against ERBB2 (#2242), phosphorylated (p-)ERBB2 (Tyr1248) (#2247), ERBB3 (C-terminus) (#12708), p-ERBB3 (Tyr1289) (#4791), ERK (#4695), p-ERK (#4370), AKT (#4685), p-AKT (#4060), GFP (#2555), Myc (#2272), and GAPDH (#5174) were purchased from Cell Signaling Technology.

### Statistical analysis

Comparisons were performed with the two-tailed Student’s t test. Results are shown as mean ± SD (*n* = 3). *P* < 0.05 was considered statistically significant.

## Results

### Identification of *ERBB3* compound variants

**Fig. 2:**
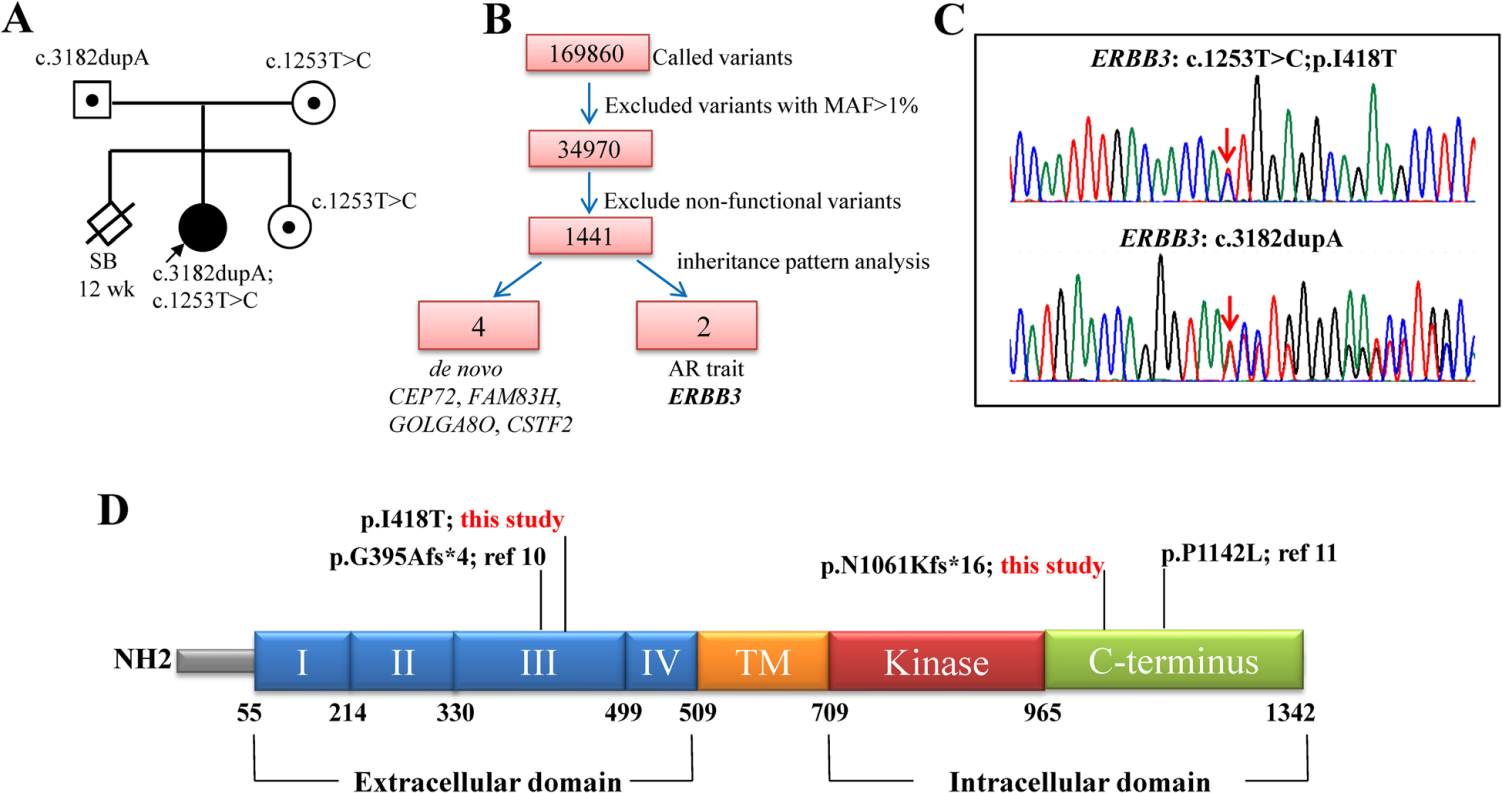
Genomic DNA sequencing of the pedigree. **a** Pedigree of the patient. **b** The data analysis algorithm used for filtering all single nucleotide variants identified using trio-based, whole exome sequencing, with the number of remaining variants after each filtering step. On filtering and prioritization, compound heterozygous variants of the *ERBB3* gene were identified as the top candidate. MAF, minor allele frequency. **c** Sanger sequencing confirmed the compound heterozygous variants, c.1253 T > C;p.I418T and c.3182dupA;p.N1061Kfs*16, in the patient. Red arrows indicate variant bases. **d** Distribution of loss-of-function germline mutations in the ERBB3 protein

Since the patient exhibited delays in postnatal growth (height/length) and development and limb asymmetry, Silver–Russell syndrome (SRS) was initially suspected. MLPA was used to detect uniparental disomy (UPD) 7/UPD14 and the methylation status of 11p15, and the results were normal (data not shown). Next, trio-WES was used to screen for the disease-causing gene (Fig. 2a and b, Additional file 3: Table S2). Candidate variants were filtered using Ingenuity software (Qiagen) according to a previously described filter strategy [15, 16]. After removing common variants (minor allele frequency > 1%) and non-functional variants, analysis under inheritance model identified four de novo variants in *CEP72*, *FAM83H*, *GOLGA8O*, and *CSTF2*, respectively, and compound heterozygous variants in *ERBB3* (Fig. 2b, Additional file 4: Table S3 and Additional file 5: Table S4). By analyzing the function of the four genes and by considering the harmless prediction results of the variants, the above four de novo variants were excluded. After prioritization, the compound heterozygous variants in the *ERBB3* gene were identified as the top candidates, which were confirmed by Sanger sequencing. One was a missense variant with a low allele frequency of 0.0032% (gnomAD database, http://gnomad.broadinstitute.org/) in exon 11 that leads to an amino acid conversion (c.1253 T > C, p.I418T) in the third subdomain of the extracellular domain. The other was a duplication of a single base (c.3182dupA) in exon 27 that was predicted to result in a frameshift leading to a premature stop codon (p.N1061Kfs*16) at the C terminus (Fig. 2c and d). Her father was heterozygous for the c.3182dupA variant, while her mother was heterozygous for the missense variant. Copy number variation (CNV) analysis was performed using WES data, and no questionable CNV was found. For the second daughter, sequencing of the *ERBB3* gene using DNA from fetal chorionic tissue at 10 weeks of pregnancy showed heterozygosity of the missense variant and an absence of the frameshift variant, which was confirmed after birth.

### In silico analysis of the p.Ile418Thr variant

**Fig. 3:**
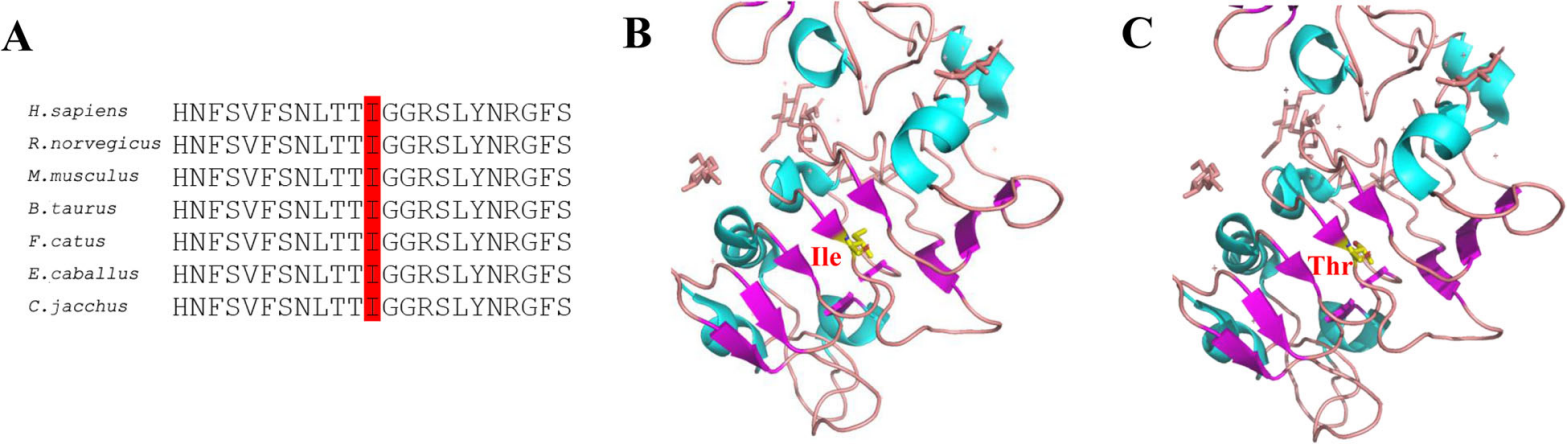
In silico analysis of the p.Ile418Thr variant. **a** Alignment of amino acid sequences among various species; the position of the mutant residue within the highly conserved region is indicated in red. **b**, **c** Homology models of the WT ERBB3 (**b**) and p.Ile418Thr mutant ERBB3 (**c**) N-terminal tails of the extracellular domain. Residue at position 418 is shown in yellow

We evaluated the pathogenicity of the p.Ile418Thr variant of *ERBB3* using a number of in silico tools. The amino acid residue at position 418 of ERBB3 is highly conserved in multiple species (Fig. 3a); the residue is located in the N-terminal tail of the extracellular domain, which participates in the formation of a β-pleated sheet (Fig. 3b, c). Functional prediction of the p.Ile418Thr variant with VarCards showed a deleterious effect on the ERBB3 protein according to SIFT (damaging, score = 0.0), PolyPhen-2 (probably damaging, score = 1.0), MutationTaster (disease causing, score = 1), PROVEAN (deleterious, score = − 4.76), and CADD (damaging, score = 27.4).

### In vitro expression analysis of *ERBB3* variants

**Fig. 4:**
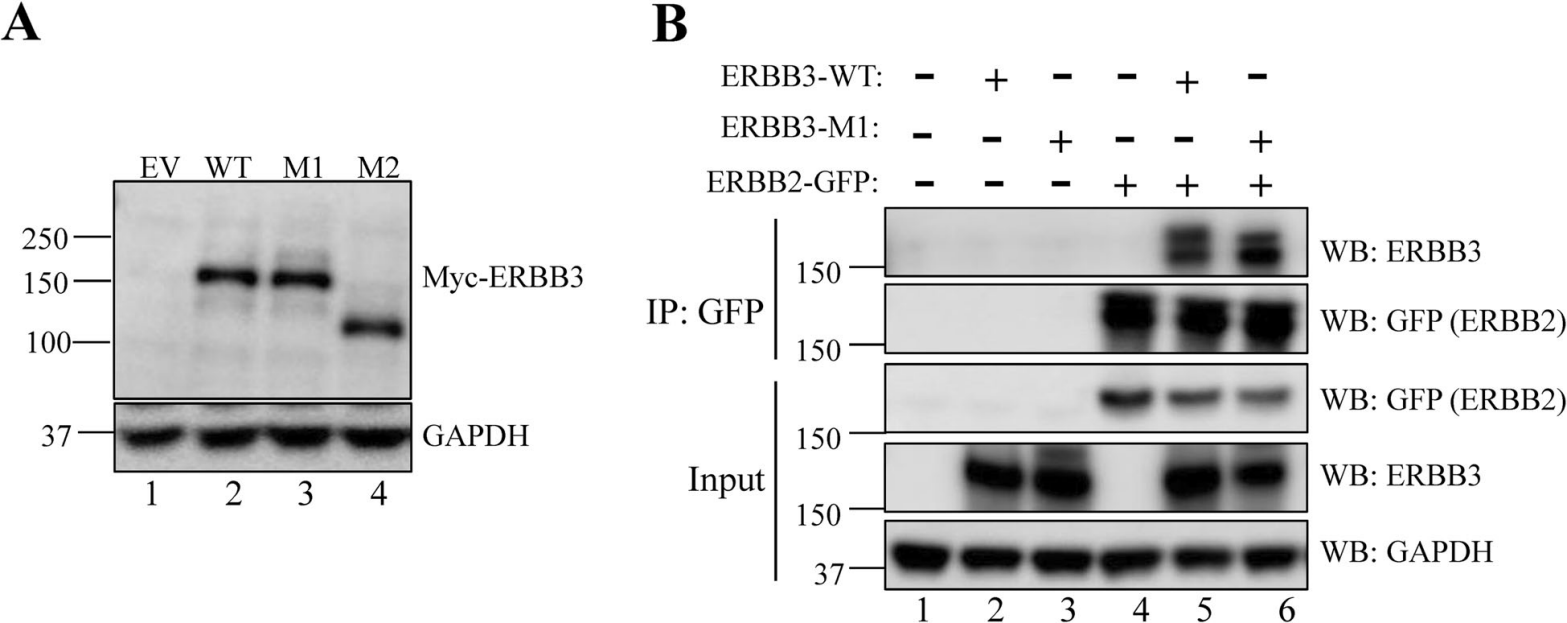
The c.1253 T > C (p.I418T) mutation has no effect on ERBB3 expression and interaction with ERBB2, whereas the c.3182dupA (p.N1061Kfs*16) mutation produces a novel truncated protein. **a** Western blotting results using anti-Myc antibody to detect ERBB3 in lysates of HEK293T cells transfected with 2 μg empty vector (EV) or WT, c.1253 T > C (M1), or c.3182dupA (M2) plasmids. **b** Results of co-immunoprecipitation to detect the interaction between ERBB2 and WT or I418T (M1) mutant ERBB3

To investigate the impact of the two variants on ERBB3 protein function, we constructed expression plasmids encoding the WT protein and the missense variant (M1: c.1253 T > C;p.I418T). The frameshift variant was predicted to undergo nonsense-mediated decay, and thus, was undoubtedly pathogenic. However, it is also possible that the variant can result in a truncated protein as it is close to the C-terminus; thus, we also constructed an expression plasmid (M2: c.3182dupA; p.N1061Kfs*16). HEK293T cells—which have no detectable ERBB3 expression [6]—were transiently transfected with the plasmids. No significant difference was observed in the expression level or molecular weight of ERBB3 between the WT and I418T variant by western blotting (Fig. 4a, lane 3). A new band, which was smaller than the WT ERBB3 band and had a size between 100 and 150 kDa, was observed for the N1061Kfs*16 variant by western blotting (Fig. 4a, lane 4). We next tested whether the I418T variant affects the interaction between ERBB2 and ERBB3 by co-transfecting HEK293T cells with ERBB2-GFP along with ERBB3-WT or ERBB3-I418T plasmids and using anti-GFP antibody-conjugated beads to pull down proteins interacting with ERBB2. Immunoblotting using an anti-ERBB3 antibody revealed that the I418T mutant protein interacted normally with ERBB2 (Fig. 4b, lane 6).

### *ERBB3* variants show reduced ERBB2/ERBB3 phosphorylation and activation of AKT and ERK signaling

**Fig. 5:**
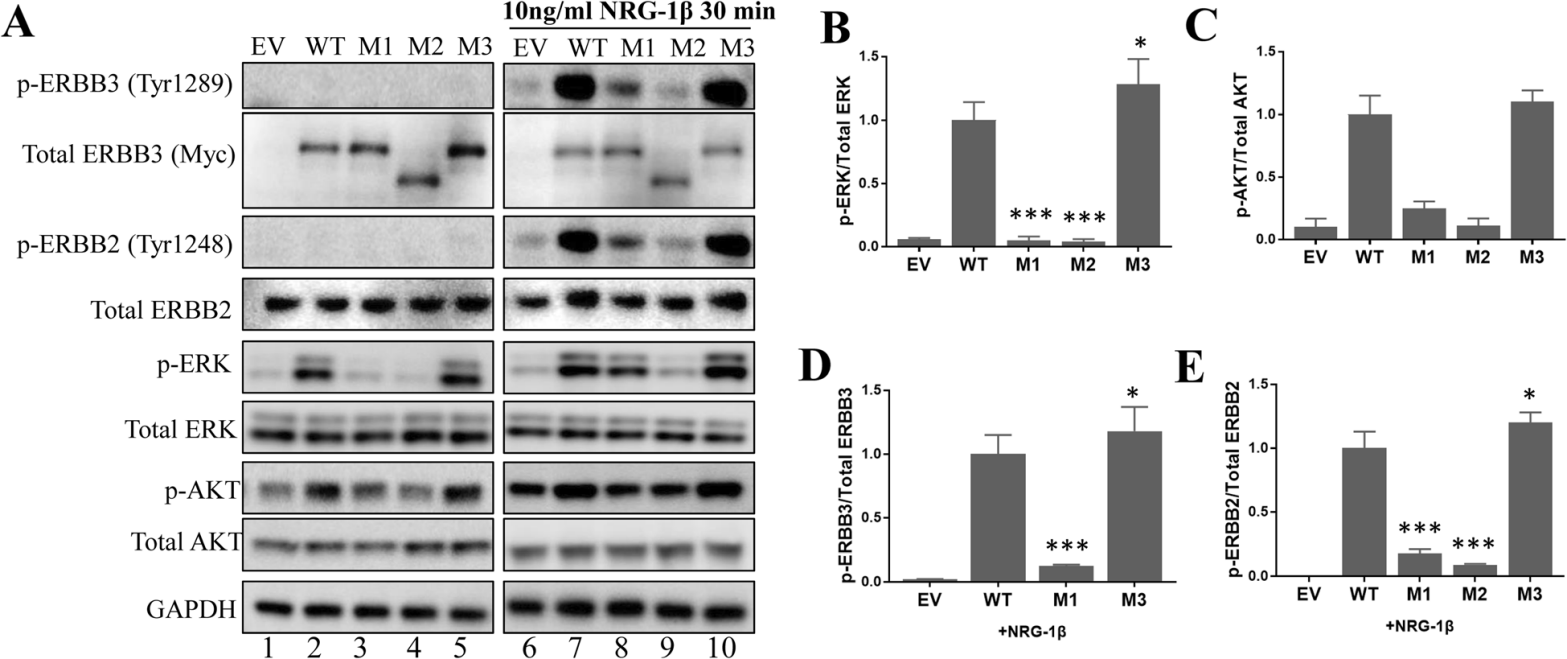
*ERBB3* variants lack the capacity to activate PI3K/AKT and ERK signaling pathways. **a** Immunoblot analysis was performed using indicated antibodies to determine the effects of WT or mutant ERBB3 on PI3K/AKT and ERK pathway activation. To induce protein phosphorylation, HEK293T cells were treated with 10 ng/ml NRG-1β for 30 min after transfection with empty vector (EV), WT, M1, M2, or M3 (V104 L) plasmids. **b–e** Quantitative analysis of p-ERK, p-AKT, p-ERBB2, and p–ERBB3 expression. *P < 0.05, **P < 0.01, ***P < 0.001 vs. WT

To clarify the pathogenic effects of the I418T and N1061Kfs*16 variants, we examined changes in the activation of signaling pathways downstream of ERBB3. As a positive control, we used a previously described gain-of-function variant (M3: c.310G > T;p.V104 L) [6, 17]. In untransfected cells, the phosphorylation of ERBB2 and ERBB3 was almost undetectable (Fig. 5a). Compared to that of the WT protein and V104 L variant, expression of ERBB3-I418T and N1061Kfs*16 variants blocked ERK and AKT phosphorylation (Fig. 5a, lanes 3 and 4 and Fig. 5b and c). In cells treated with NRG-1β, the ERBB3-I418T protein was not fully phosphorylated (Fig. 5a, lane 8, and Fig. 5d). We were unable to detect N1061Kfs*16 protein phosphorylation due to the unavailability of a commercial antibody recognizing the phosphorylation site before N1061. Neither of the variants induced ERBB2 expression; moreover, they blocked the phosphorylation of ERBB2 protein (Fig. 5a, lanes 8 and 9, and Fig. 5e), thereby decreasing p-ERK and p-AKT levels.

## Discussion

ERBB2 is activated through heterodimerization with other EGFR family members; however, it fails to bind to HRG; instead, it depends on HRG to bind to ERBB3 or ERBB4. The ERBB2/ERBB3 heterodimer functions as an oncogenic unit that drives tumorigenesis; for instance, ERBB3 phosphorylation is increased in many ERBB2-overexpressing breast tumors [18–20]. Additionally, *ERBB2* and *ERBB3* gain-of-function mutations have been shown to contribute to the occurrence and development of a variety of cancers [17]. However, there is limited knowledge of the loss-of-function phenotype of ERBB3 in humans. In this study, we report for the first time that germline loss-of-function variants of the *ERBB3* gene are associated with the development of multiple congenital deformities. We identified compound heterozygous variants (c.1253 T > C;p.I418T and c.3182dupA;p.N1061Kfs*16) in the *ERBB3* gene; the former had no impact on ERBB3 expression or interaction with ERBB2 protein, but impaired ERBB2 and ERBB3 phosphorylation and blocked the activation of AKT and ERK pathways, whereas the latter truncated protein that was also unable to activate downstream signaling. The following two points may explain why the ERBB3 (p.I418T)/ERBB2 heterodimer fails to induce signaling. Firstly, although the missense variant has no significant impact on the secondary structure of the protein (Fig. 3), it might alter the asymmetrical interaction of the heterodimeric complex, which is crucial for downstream signaling activation. It has been observed that the catalytically inactive ERBB3 can still be an ‘active’ participant in a heterodimeric complex [21, 22]. Secondly, ERBB3 possesses direct binding sites for the p85 subunit of PI3K, allowing activation of PI3K and its downstream signaling components independent of interaction with ERBB2 [23]. Thus, inactive ERBB3 may directly impair downstream signaling activation.

ERBB3 plays a critical role in early embryonic development in mice. *Erbb3*^+/−^ mice were healthy and fertile, while *Erbb3*^*−*/−^ mice were embryonic lethal at embryonic day (E)11.5 to E13.5 due to a lack of connective tissue in the atrioventricular valves, leading to blood reflux [24, 25]. These embryos also showed severe anomalies in brain development including stagnation of cerebellar development, an absence of Schwann cell precursors, mandibular division of the trigeminal nerve, and abnormal development of stomach, pancreas, and adrenal glands. In the Israeli Bedouin kindred pedigree, 17 patients died from soon after birth to 1 year old, possibly due to a complete lack of functional ERBB3 expression. In contrast, the I418T variant in our patient showed partial functional expression, which ensured a certain degree of growth and development.

To date, only two homozygous variants of the *ERBB3* gene have been reported to cause Mendelian phenotypes in humans (Fig. 2c) [11, 12]. Due to a lack of detailed clinical information in the second report [12], we mainly compared the phenotypes of our patient to those observed in the Israeli Bedouin kindred pedigree [11]. Although there were some shared features such as cardiac malformation and ophthalmologic problems, several differences were observed (Table 1). Firstly, all 23 patients in the Israeli Bedouin kindred pedigree had multiple joint contracture and two had hip dislocation, while the patient reported by Alfares et al. also had hip and knee dislocation [12], suggesting that joint deformities arise from *ERBB3* variation. However, our patient did not show any joint deformity. Secondly, half of the patients (12/23) in the earlier report had an enlarged urinary bladder and five exhibited kidney malformation, which were also absent in our patient. Thirdly, our patient showed more serious facial malformations along with developmental delay, asymmetrical limbs, immunodeficiency, liver damage, feeding difficulties, and anemia, which have not been previously described. The definite molecular mechanism by which ERBB3 maintains the normal growth and development of different organ tissues and cells remains unclear. Previous studies on the two ERBB3-related contractures as well as the murine knockout model also did not reveal any such mechanism. Considering the widespread expression of ERBB3 and the importance of AKT and ERK signaling in development, different mechanisms are very likely to exist in different organ tissues and cells. *ERBB3* is known to primarily function as an oncogene for the activation of PI3K/AKT and ERK signaling to promote tumor cell proliferation or differentiation; hence, we believe that the inactivation of these pathways might limit cell proliferation, leading to dysplasia of multiple organs. Meanwhile, prolonged activation of PI3K/AKT and ERK signaling disrupts the regulation of cell growth and division, leading to the characteristic features of Noonan syndrome (OMIM# 163950) that is caused by germline variation in *KRAS* genes (*PTPN11*, *SOS1*, *RAF1*, *LZTR1*, etc.) [26]. Therefore, we assume that organ development requires the appropriate expression of these two pathways. Both over-activation and inactivation of these pathways will result in a complex phenotype. Moreover, the 23 patients reported by Landau et al. harbored the same homozygous variant, but showed variable phenotypes. Differences in the proportion of the mutation in different tissues might also be a cause of the phenotypic differences in these patients.

Our patient was suspected of having SRS at 7 months of age mainly based on lower limb asymmetry, as well as postnatal growth failure, protruding forehead, feeding difficulties, and a low BMI [27]. However, the lower limb asymmetry had almost completely recovered at 24 months of age. Moreover, she was not small-for gestational age (birth weight at − 1.9 SD), which is a primary feature of SRS; however, we could not obtain birth head circumference data for the patient, making it very difficult to assess the patient using the SRS criteria. Therefore, we prefer to define the patient’s condition as novel multisystem syndrome without congenital contracture.

Taking into consideration the substantial phenotype differences between our patient and the previously reported cases, we performed a trio-whole genome sequencing for the patient in an attempt to identify possibly detrimental intronic variants implicated in development disorders. However, no unexpected intronic variants were found (data not shown). Identification of more cases with *ERBB3* loss-of-function variants and similar phenotypes will be helpful to establish the genotype-phenotype relationship in the future studies. Moreover, further studies should consider using animal models with the specific variant (I418T) identified in the patient and try to rescue the phenotype with wild type *ERBB3* cDNA, rather than the mutant cDNA, in ERBB3-deficient cells derived from patients or animal models. Generation of an I418T knock-in cell line, for example in U2OS cells, is also necessary to study the molecular mechanism.

## Conclusions

In summary, we reported the first patient with a novel multisystem syndrome associated with novel compound heterozygous variants of the *ERBB3* gene. The in vitro functional results suggested that loss of function of ERBB3 is related to this human phenotype. It implies that the spectrum of features associated with *ERBB3* variation is broader than previously thought, and provides new evidence that biallelic loss of function variants of *ERBB3* may contribute to a developmental disorder involving multiple organ systems.

## Supplementary information

**Additional file 1: Figure S1.** X-ray results of the patient.

**Additional file 2: Table S1.** Primers for the *ERBB3* (NM_001982.3) mutant plasmids construction.

**Additional file 3: Table S2.** The quality of WES data.

**Additional file 4: Table S3.** Biological filtering by de novo model.

**Additional file 5: Table S4.** Biological filtering by ‘recessive inheritance’ model.

## Data Availability

The whole exome sequencing data that support the findings of this study are available on request from the corresponding author.

## Abbreviations

ASD: Atrioventricular septal defect
BMI: Body mass index
ERBB3: Erb-B2 receptor tyrosine kinase 3
ERK: Extracellular signal-regulated kinase
GAPDH: Glyceraldehyde 3-phosphate dehydrogenase
Het: Heterozygote
Hom: Homozygote
HRG: Heregulin
Ig: Immunoglobulin
MLPA: Multiplex ligation-dependent probe amplification
NRG: Neuregulin
PI3K: Phosphatidylinositol 3-kinase
SRS: Silver–Russell syndrome
VSD: Ventricular septal defect
WES: Whole exome sequencing
