# Supplementary figures and images for: Biallelic ERBB3 loss-of-function variants are associated with a novel multisystem syndrome without congenital contracture
Source: Orphanet J Rare Dis. 2019 Nov 21;14:265. doi: 10.1186/s13023-019-1241-z (PMC6868814; doi:10.1186/s13023-019-1241-z)

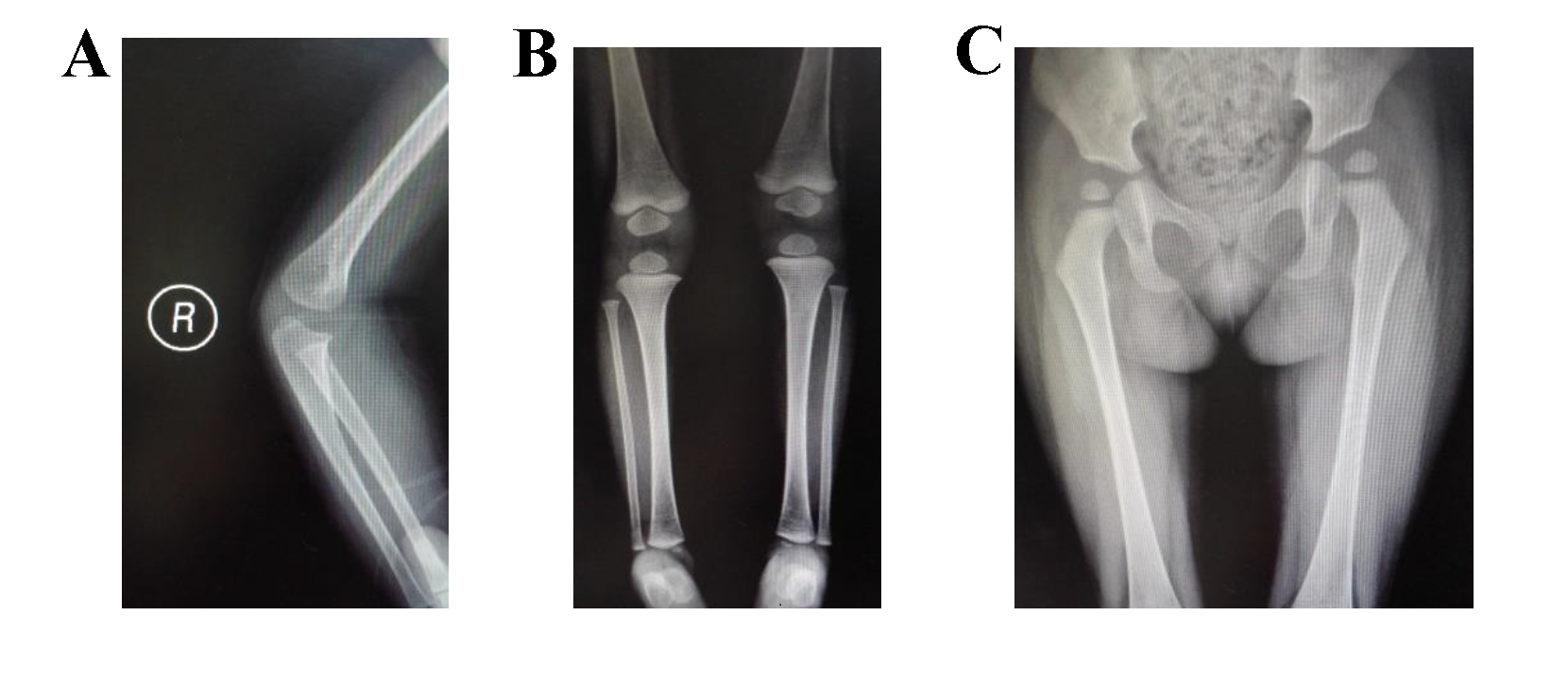

Supplement: Supplementary file 1 — Additional file 1: Figure S1. X-ray results of the patient. [file 13023_2019_1241_MOESM1_ESM.tiff]
