# Supplementary material for: Biallelic ERBB3 loss-of-function variants are associated with a novel multisystem syndrome without congenital contracture
Source: Orphanet J Rare Dis. 2019 Nov 21;14:265. doi: 10.1186/s13023-019-1241-z (PMC6868814; doi:10.1186/s13023-019-1241-z)
Supplement: Supplementary file 2 — Additional file 2: Table S1. Primers for the ERBB3 (NM_001982.3) mutant plasmids construction. [file 13023_2019_1241_MOESM2_ESM.docx]

**Table S1. Primers for the *ERBB3* (NM_001982.3) mutant plasmids construction.**

|  | Forward primer 5’-3’ | Backward primer 5’-3’ |
| --- | --- | --- |
| M1: c.1253T>C | TTGACAACCACTGGAGGCAGAAGCCT | CTGCCTCCAGTGGTTGTCAAATTGG |
| M2: c.3182dupA | CCAGGGTAAATCTTGGGGAGTCTTGCCAGG | TCCCCAAGATTTACCCTGGTTCATGGGCATG |
| M3: c.310G>T | CAACCTCCGCTTGGTGCGAGGGA | TCCCTCGCACCAAGCGGAGGTTG |
