# Supplementary material for: Biallelic ERBB3 loss-of-function variants are associated with a novel multisystem syndrome without congenital contracture
Source: Orphanet J Rare Dis. 2019 Nov 21;14:265. doi: 10.1186/s13023-019-1241-z (PMC6868814; doi:10.1186/s13023-019-1241-z)
Supplement: Supplementary file 3 — Additional file 3: Table S2. The quality of WES data. [file 13023_2019_1241_MOESM3_ESM.docx]

**Table S2. The quality of WES data**

|  |  |  | **Patient** | **Father** | **Mother** |
| --- | --- | --- | --- | --- | --- |
| Total Reads |  |  | 108590635 | 70064782 | 99662440 |
| Aligned Reads |  |  | 97.995% | 98.415% | 98.601% |
| Reads on Target |  |  | 84.213% | 82.224% | 82.327% |
| Average Coverage |  |  | 197.98× | 123.90× | 178.46× |
| Percent of ROI with 1× |  |  | 99.604% | 99.712% | 99.600% |
| Percent of ROI with 20× |  |  | 97.416% | 95.538% | 97.368% |
| Uniformity |  |  | 98.59% | 98.78% | 98.66% |

ROI: regions of interest
