# Supplementary material for: Biallelic ERBB3 loss-of-function variants are associated with a novel multisystem syndrome without congenital contracture
Source: Orphanet J Rare Dis. 2019 Nov 21;14:265. doi: 10.1186/s13023-019-1241-z (PMC6868814; doi:10.1186/s13023-019-1241-z)
Supplement: Supplementary file 4 — Additional file 4: Table S3. Biological filtering by de novo model. [file 13023_2019_1241_MOESM4_ESM.docx]

| Chromosome | Position | Gene Symbol | Transcript Variant | Protein Variant | Aelle frequency (gnomAD) | Function Prediction (Score) |
| --- | --- | --- | --- | --- | --- | --- |
| 5 | 633939 | CEP72 | c.568A>C | p.M190L | 0 | SIFT: Tolerated (0.361)  PolyPhen-2: Benign (0.006)  CADD: Tolerable (5.897) |
| 8 | 144810231 | FAM83H | c.1400C>T | p.A467V | ALL:0.0009% | SIFT: Damaging (0.028)  PolyPhen-2: Benign (0.052)  CADD: Tolerable (9.189) |
| 15 | 32743123 | GOLGA8O | c.583delG | p.A195fs*16 | 0 |  |
| X | 100075437 | CSTF2 | c.32T>C | p.V11A | 0 | SIFT: Tolerated (0.2)  PolyPhen-2: Benign (0.002)  CADD: Damaging (22.4) |

**Table S3. Biological filtering by *de novo* model**
