# Supplementary material for: Biallelic ERBB3 loss-of-function variants are associated with a novel multisystem syndrome without congenital contracture
Source: Orphanet J Rare Dis. 2019 Nov 21;14:265. doi: 10.1186/s13023-019-1241-z (PMC6868814; doi:10.1186/s13023-019-1241-z)
Supplement: Supplementary file 5 — Additional file 5: Table S4. Biological filtering by ‘recessive inheritance’ model. [file 13023_2019_1241_MOESM5_ESM.docx]

| Chromosome | Position | Gene Symbol | Genetic model | Transcript Variant | Protein Variant | Aelle frequency (gnomAD) | Origin | Function Prediction |
| --- | --- | --- | --- | --- | --- | --- | --- | --- |
| 12 | 56486839 | ERBB3 (NM_001982.3) | AR | c.1253T>C (het) | p.I418T | ALL:0.0032% | Mother | SIFT: Damaging PolyPhen-2: Probably Damaging |
| 12 | 56494010 | ERBB3 (NM_001982.3) |  | c.3182dupA (het) | p.N1061fs*16 | 0 | Father |  |

**Table S4. Biological filtering by ‘recessive inheritance’ model**

Het, heterozygous; AR, autosomal recessive
